# A long-tailed marine reptile from China provides new insights into the Middle Triassic pachypleurosaur radiation

**DOI:** 10.1038/s41598-022-11309-2

**Published:** 2022-05-05

**Authors:** Guang-Hui Xu, Yi Ren, Li-Jun Zhao, Jun-Ling Liao, Dong-Hao Feng

**Affiliations:** 1 Key Laboratory of Vertebrate Evolution and Human Origins of Chinese Academy of Sciences, Institute of Vertebrate Paleontology and Paleoanthropology, Chinese Academy of Sciences, Beijing, 100044 China; 2 CAS Center for Excellence in Life and Paleoenvironment, Beijing, 100044 China; 3 University of Chinese Academy of Sciences, Beijing, 100049 China; 4 Zhejiang Museum of Natural History, Hangzhou, 310014 China; 5 College of Resource and Environmental Engineering, Key Laboratory of Karst Georesources and Environment, Ministry of Education, Guizhou University, Guiyang, 550025 China; 6 College of Economics and Management, Xingyi Normal University for Nationalities, Xingyi, 562400 China

**Keywords:** Palaeontology, Taxonomy

## Abstract

Pachypleurosaurs (Pachypleurosauroidea) are a group of small to medium-sized, lizard-like marine reptiles in the Early to Middle Triassic, including Pachypleurosauridae, Keichousauridae and closely related taxa. The group is generally considered as a sauropterygian radiation, but its phylogenetic interrelationships remain highly debated. Here, we present a new pachypleurosaurid, *Honghesaurus longicaudalis* gen. et sp. nov., from the early Middle Triassic (Anisian, ~ 244 Ma) marine deposits in Luxi, Yunnan, China. The discovery documents the first really long-tailed pachypleurosaur with totally 121 (69 caudal) vertebrae, providing new evidence for the vertebral multiplication and ecological adaption of this group. The long trunk associated with an incredibly long tail could provide *Honghesaurus* the advantage of maneuverability and energy efficiency for lateral undulatory swimming. *Honghesaurus*, although possessing a series of autapomorphies, fills the morphological gap between *Qianxisaurus* from the Ladinian Xingyi Biota and *Wumengosaurus* from the Anisian Panxian Biota. Phylogenetic studies unite these three pachypleurosaurids as a monophyletic clade above European pachypleurosaurid clades and provide new insights into the interrelationships of this group. Our scenario of pachypleurosaurian phylogeny combined with the stratigraphic data imply that the Tethys Ocean was a west–east corridor for dispersal of pachypleurosaurids from Europe into South China.

## Introduction

Reptiles are a primarily terrestrial assemblage, but several groups adapted the marine environments in the aftermath of the end-Permian extinction, including Ichthyosauria, Thalattosauria and Sauropterygia^1–7^. Among them, the Sauropterygia is the largest, most successful group of marine reptiles with more than 180 species in about 120 genera recovered, spanning from the late Early Triassic to the Late Cretaceous (~ 245–65.5 Ma)^8,9^. The group exploited a wide range of habitats and ecological niches, and diversified into the durophagous placodonts (including unarmoured or partly armoured Placodontoidea and strongly armoured Cyamodontoidea), the shallow marine pachypleurosaurs (Pachypleurosauroidea) and nothosaurs, and the obligate swimming plesiosaurs^8–11^. The pachypleurosaurs are lizard-like marine reptiles in the Early to Middle Triassic^12–31^. They have been considered to be a monophyletic group of eosauropterygian radiation within the Sauropterygia, although different views exist^23–35^. In general, pachypleurosaurs remain plesiomorphic for sauropterygians and have long attracted the attention of palaeontologists interested in the early evolution of this clade^12–26^. However, the interrelationships of pachypleurosaurs are still controversial, hampering our understanding on their evolutionary and palaeobiogeographic history.

In the Triassic, almost all of the Earth’s landmasses were combined in the supercontinent of Pangaea, surrounded by a vast ocean of Panthalassa. An arm of this ocean, namely the Tethys (including the Palaeo-Tethys in the north and the Neo-Tethys in the south), intruded deeply into the centre of Pangaea at the equator^36,37^. Pachypleurosaurs are known only from the Tethys ocean, and its potential diet may include soft-bodied invertebrates (e.g., cephalopods), some shrimps, and small or juvenile fishes^38^. The earliest and basal pachypleurosaurs can be traced to the Early Triassic in the eastern Tethys realm (represented by *Majiashanosaurus*^21^ from Chaohu, Anhui, South China), and in the Middle Triassic, this clade underwent a rapid radiation, represented heretofore by eight genera from the eastern Tethys and five from the western Tethys (Europe). The unnamed pachypleurosaur recovered from Myanmar^39^, represented by two incomplete, poorly preserved specimens from the Nwabangyi Dolomite Formation (likely Early to Middle Triassic), is an interesting record of this clade outside China in the eastern Tethys realm, but it is currently hard to be incorporated into a phylogenetic analysis because of insufficient anatomical data and its age needs a further constraint. It is generally accepted that all pachypleurosaurs from the western Tethys are members of the family Pachypleurosauridae, but those in the eastern Tethys are more complicated in diversity and controversial in taxonomy, and resolution of their relationships are critical for understanding the origin and evolution of this group. The Chinese pachypleurosaurs include Keichousauridae and other pachypleurosaurid-like forms^20–31^. Among them, *Keichousaurus* is one of the most abundant marine reptiles from the late Middle Triassic (Ladinian) of China. It was originally classified in the Pachypleurosauridae but later in its own family Keichousauridae^18^. Phylogenetic studies recover the Keichousauridae (*Keichousaurus* and *Dianopachysaurus*) either as the sister group of the Pachypleurosauridae^20^ or as a basal lineage of the Nothosauroidea^24^. Additionally, *Wumengosaurus* from the Middle Triassic Panxian Biota in Guizhou is the most taxonomically problematic taxon among Chinese pachypleurosaurid-like forms; the genus has been considered as either a pachypleurosaurid^23,31^ or a basal member of the clade Nothosauroidea plus Pachypleurosauridae^25^. Another hypothesis that places *Wumengosaurus* as a close relative of the Ichthyopterygia and Hupehsuchia has also been proposed^40^, but it is very weakly supported and has rarely been accepted.

**Figure 1:**
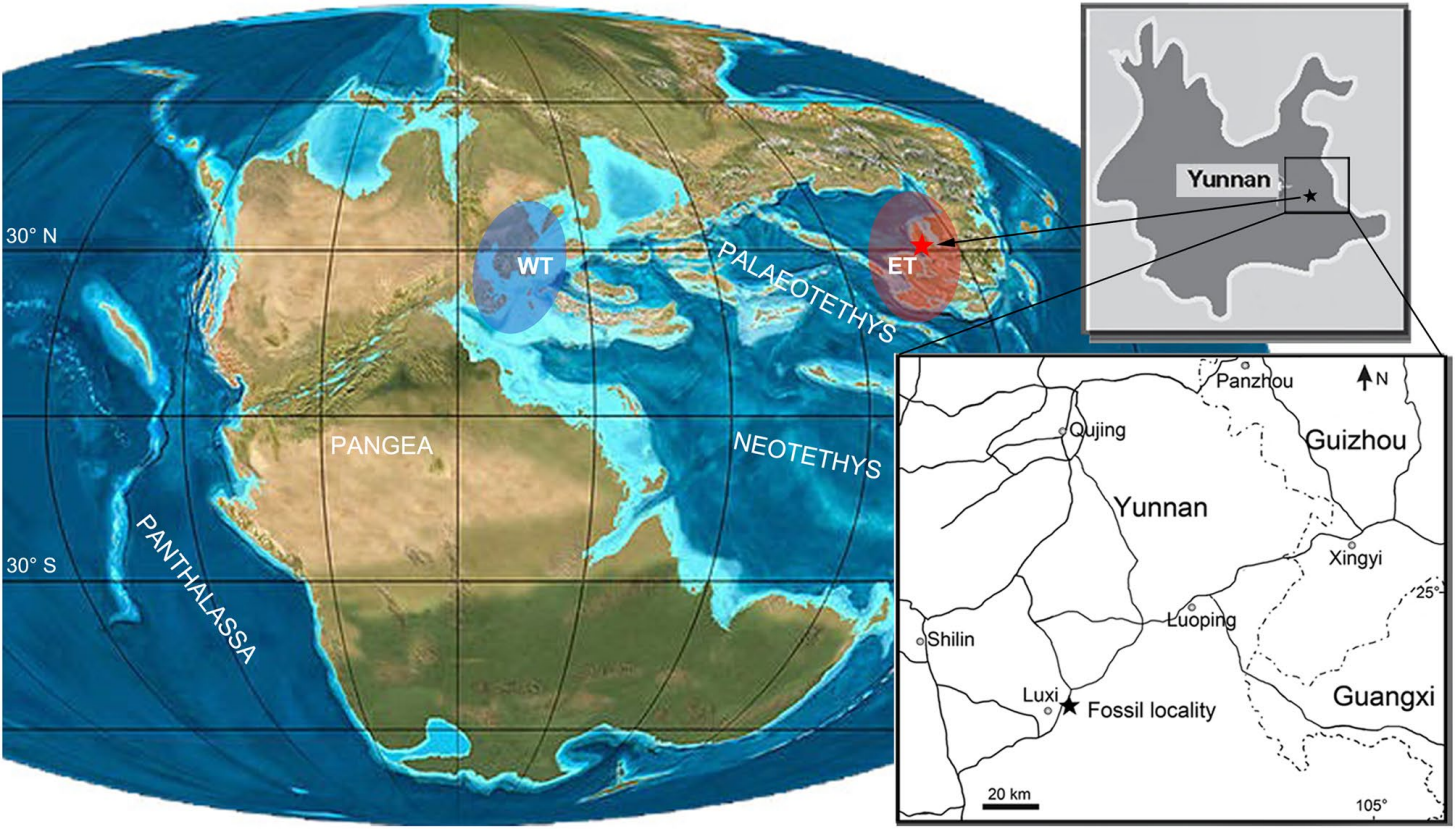
Locality map. The fossil locality is indicated by a star. The Middle Triassic (Anisian) palaeogeography is modified from ref.^37^, with the eastern Tethyan realm (ET, red) and western Tethyan realm (WT, blue) highlighted.

Here, we report the discovery of a *Wumengosaurus-*like pachypleurosaur based on a specimen from the Second (Upper) Member of the Guanling Formation exposed in Luxi, Yunnan, South China (Fig. 1). The specimen was collected in 2021 during the autumn fieldwork led by the first author. The fossil skeleton preserved in a large slab (350 mm × 605 mm) of micritic limestone is fully exposed after 2 months’ preparation by the first and last authors and their colleague (Meng-Nan Lü). Impressively, the specimen is exceptionally preserved, representing one of the most complete skeletons of pachypleurosaurs from the Middle Triassic Luoping Lagerstätte or Biota. This Lagerstätte, renowned by its superb preservation and taxonomic richness (including abundant invertebrates, fishes, marine reptiles and plants), was originally found in Luoping of Qujing, and later fieldworks extended its distribution into the nearby Luxi of Honghe, eastern Yunnan^41–44^. A detailed geological survey indicates that the fossiliferous level of the Luoping Biota (middle part of the Second Member of the Guanling Formation) is slightly lower than that of the Panxian Biota (upper part of the Second Member of the Guanling Formation), although both are located at the same stage (Pelsonian, Anisian) of the Middle Triassic by the conodont analyses^45–47^. The fossil beds are composed of thinly laminated micritic limestone alternating with silty limestone, indicating a semi-enclosed intraplatform depositional environment in the early Middle Triassic Yangtze Sea, a part of the eastern Palaeotethys Ocean^41,42^.

## Results

### Systematic paleontology

Sauropterygia Owen, 1860^48^.

Eosauropterygia Rieppel, 1994^9^.

Pachypleurosauroidea Huene, 1956^49^.

Pachypleurosauridae Nopcsa, 1928^50^.

*Honghesaurus longicaudalis* gen. et sp. nov.

*Etymology* The genus name refers to Honghe Prefecture, where the holotype was located; the species epithet is derived from *longi* plus *caudalis* (Latin for long tail), referring to its incredibly long tail.

*Holotype* A complete skeleton in the collection of the Institute of Vertebrate Paleontology and Paleoanthropology, Chinese Academy of Sciences (IVPP V30380).

*Locality and horizon* Luxi, Honghe, Yunnan, China; Second (Upper) Member of Guanling Formation, Pelsonian (~ 244 Ma), Anisian, Middle Triassic.

*Diagnosis* A pachypleurosaurid distinguishable from other members of this family by the following autapomorphies: snout longer than postorbital portion of skull, 47.8% of skull length; external naris longitudinally retracted, 47.7% of orbital length; supratemporal fossa oval, 46.2% of orbital length; about ten teeth in anteriorly pointed premaxilla; two fossae in retroarticular process; 20 cervical, 29 dorsal, three sacral, and 69 caudal vertebrae; single ossified carpal; and phalangeal formula 2-3-2-4-1 for manus and 2-3-4-5-2 for pes.

**Figure 2:**
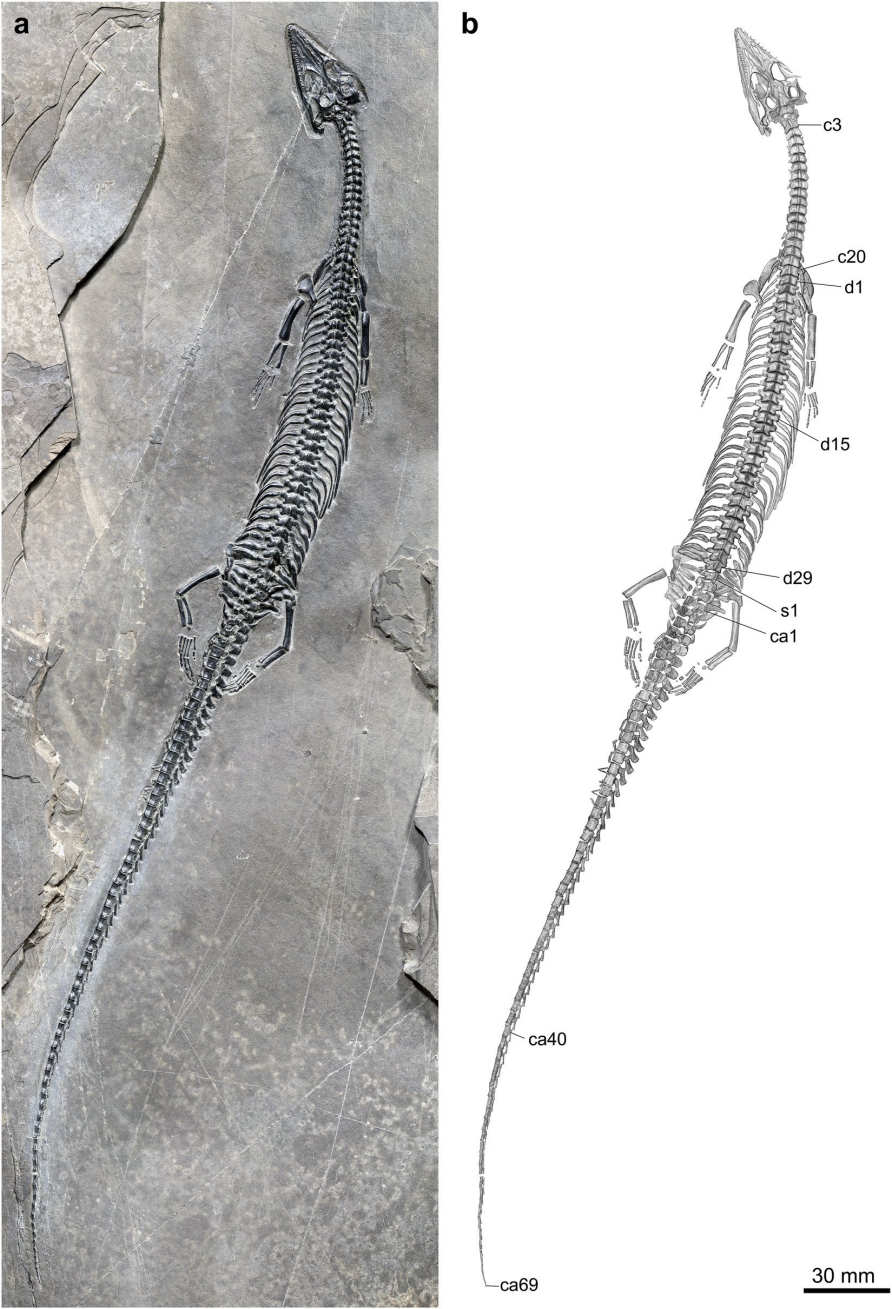
*Honghesaurus longicaudalis* gen. et sp. nov., Holotype (IVPP V30380). Photo (**a**) and line-drawing (**b**) of whole specimen. c, cervical vertebra; ca, caudal vertebra; d, dorsal vertebra; s, sacral vertebra.

**Figure 3:**
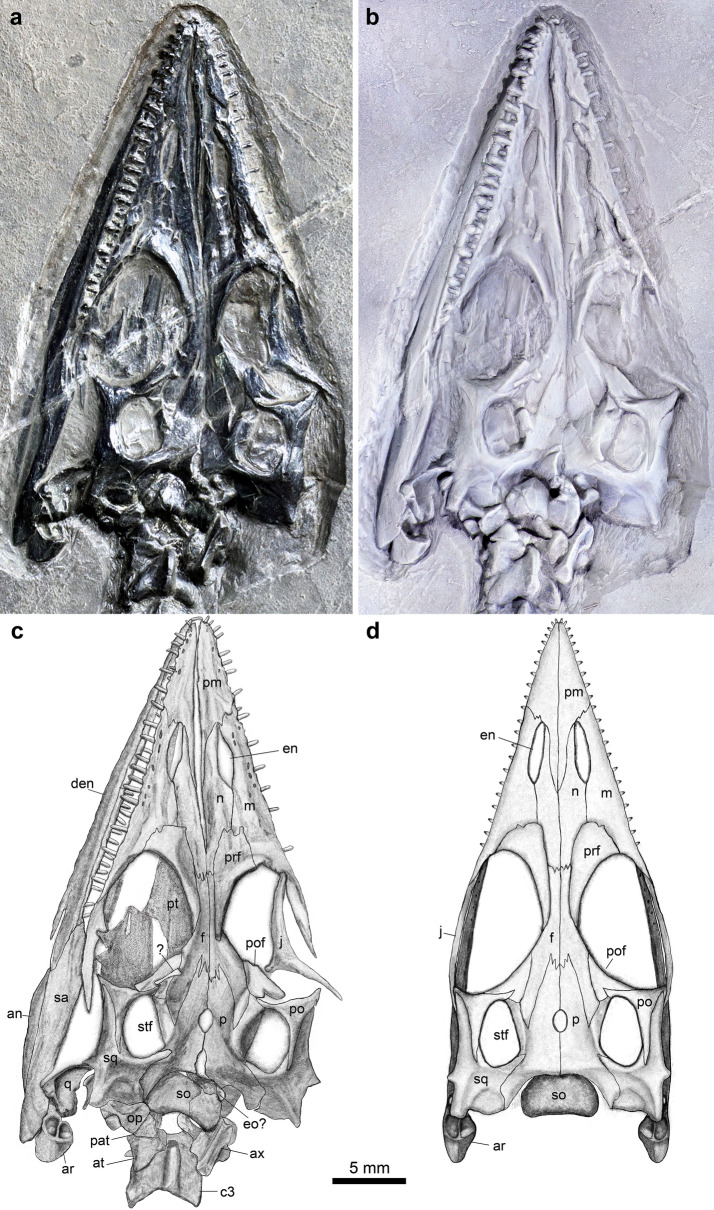
Skull and mandible of *Honghesaurus longicaudalis* gen. et sp. nov., IVPP V30380. Photo before (**a**) and after (**b**) dusted with ammonium chloride. (**c**) Line- drawing. (**d**) Reconstruction in dorsal view. an, angular; ar, articular; at, atlas; ax, axis; c, cervical vertebra; den, dentary; en, external naris; eo, exoccipital; f, frontal; j, jugal; m, maxilla; n, nasal; op, opisthotic; p, parietal; pat, proatlas; pm, premaxilla; po, postorbital; pof, postfrontal; prf, prefrontal; pt, pterygoid; q, quadrate; sa, surangular; so, supraoccipital; sq, squamosal; stf, supratemporal fossa.

*Comparative description* The holotype and only currently known specimen of *Honghesaurus* is 47.1 cm in total length. From its body size, *Honghesaurus* is in accordance with most of other pachypleurosaurs that are small-sized with a maximum total length rarely exceeding 50 cm. However, three pachypleurosaurs are notably larger, i.e., *Diandongosaurus* cf. *acutidentatus*^35^ (88 cm), *Neusticosaurus edwardsii* (120 cm) and *Wumengosaurus delicatomandibularis* (~ 130 cm). In general morphology, the most striking feature of *Honghesaurus* is its incredibly long tail, which measures 117% of the precaudal length (Table 1). The whole vertebral column consists of 121 vertebrae (Fig. 2; see descriptions below), documenting the largest number known in this group. The snout is more anteriorly pointed than most of other pachypleurosaurs (except *Wumengosaurus*). In addition, the external naris in the snout is unusually retracted (Fig. 3), resembling the conditions in *Wumengosaurus* and *Qianxisaurus*; by contrast, the external naris is commonly oval-shaped (not retracted) in other pachypleurosaurs.

**Table 1:** Measurements (in mm) of the holotype (IVPP V30380) of *Honghesaurus longicaudalis* gen. et sp. nov.

Total length	471.0
Snout length	15.8
Skull length (premaxillary symphysis to occipital condyle)	33.0
Length of external naris	4.2
Length of orbit	8.8
Length of axis to last cervical vertebra	53.6
Length of axis to last dorsal vertebra	168.7
Length of mandible	38.9 (L)
Length of humerus	15.5 (L)
Proximal width of humerus	2.9 (L)
Distal width of humerus	3.9 (L)
Length of radius	9.1 (L)
Length of ulna	8.6 (L)
Length of metacarpal I	2.2 (L)
II	3.9 (L)
III	4.5 (L)
IV	4.2 (L)
V	2.9 (L)
Length of femur	16.7 (L)
Proximal width of femur	3.4 (L)
Distal width of femur	2.7 (L)
Length of fibula	9.9 (L)
Length of tibia	9.6 (L)
Length of metatarsal I	2.9 (L)
II	5.5 (L)
III	6.4 (L)
IV	5.3 (L)
V	4.4 (L)

L, left.

The snout portion anterior to the orbit is longer than the postorbital portion of the skull, measuring 47.8% of the skull length (the premaxillary symphysis to the occipital condyle). The paired premaxillae are nearly triangular, and each bears a long posterodorsal process that sharply inserts between the anterior parts of nasals and tapers off to a point at the level of posterior margin of the external naris (Fig. 3). This process is separated from the frontal by the posterior parts of nasals which meet along the median line of the skull. The nasal, slightly longer than the premaxilla, posteriorly contacts the frontal in an interdigitating suture. The maxilla contacts the premaxilla at the anterior margin of the external naris and bears a short anteroventral process extending below the premaxilla. The large triangular ascending process of the maxilla medially inserts between the nasal and the prefrontal. Posteriorly, the maxilla bears a fairly long posterolateral process underling the anterior two-thirds of the jugal. The length of the retracted external naris is 47.7% of the orbital length, being about a quarter of the snout length. The lateral border of the naris is defined by the maxilla, and its medial border mainly by the nasal with little contribution by the premaxilla. This condition is otherwise present in *Qianxisaurus*; in other pachypleurosauroids, however, the premaxilla contributes considerably to the medial border of the external naris.

The paired frontals each has a relatively narrow and elongate main body with a large posterolateral wing inserting into the parietal posteriorly. The lateral margin of the frontal contacts the crescent-shaped prefrontal anteriorly and the nearly triangular postfrontal posteriorly, and contributes to a small part of the medial border of the large and oval orbit (measuring 26.6% of the skull length). The postfrontal tapers anteriorly and ventrally, and bears a notch in its posterior margin receiving the dorsal process of the triradiate postorbital. The descending process of the postorbital forms the ventral part of the posterior border of the orbit, and its posterior process sharply inserts the squamosal and contributes to the lateral border of the nearly oval supratemporal fenestra (measuring 46.2% of the orbital length). The jugal is open L-shaped, and forms the lateral border of the orbit. Posteriorly, the bone extends beyond the ventral margin of the postorbital and would reach the anterior process the squamosal (Fig. 3). A separate lacrimal is absent, as commonly in other sauropterygians.

The paired parietals are fairly massive. The middle portion of the parietal is concave laterally, defining the medial border of the supratemporal fenestra. Anteriorly, the parietal contacts the frontal in a zig-gag suture and the postfrontal in a nearly straight suture. Posteriorly, the bone forms a slightly concave occipital edge, and bears a long posterolateral process that contacts the posteromedial extension of the squamosal laterally. The median pineal foramen between parietals is relatively large and oval, located slightly anterior to the level of the parietal center, similar to the conditions in *Qianxisaurus* and European pachypleurosaurs (e.g., *Neusticosaurus*, *Serpianosaurus* and *Odoiporosaurus*). The relatively broad supraoccipital forms the posterior roof of the braincase. It is somewhat heart-shaped in dorsal view, lacking a median ridge. Other visible elements of the braincase include the paroccipital portion of the opisthotic-exoccipital complex, but the complete shape of this complex is still unknown.

The squamosal is large, bearing long medial and anterior processes and a short descending process. The medial process forms the main part of the posterior border of the temporal fossa and inserts into the parietal. The anterior process sutures the postorbital, and together with it, forms the bar between the supratemporal fossa and the ventrally open infratemporal fenestra. The descending process is short and triangular, well separated from the condyle of the quadrate. This resembles the condition in *Wumengosaurus*. In other pachypleurosaurids, however, the descending process of the squamosal extends further ventrally, and nearly reaches the condyle of the quadrate. The left quadrate is well exposed in lateral view with its dorsal process extending underneath the base of the descending process of the squamosal (Fig. 3). The posterior margin of the quadrate is excavated. Besides a strong mandibular condyle, the quadrate has a posteriorly projecting head, which forms the base of the posterior quadrate notch. A similar condition is also present in European pachypleurosaurids (e.g., *Pachypleurosaurus* and *Serpianosaurus*). No distinct quadratojugal is discernable associated with the quadrate.

The dentary is long and wedge-shaped, extending posteriorly to the posterior margin of the orbit. The posteroventral margin of the dentary is distinctly notch which would accommodate the anterior tip of the angular (not fully exposed; Fig. 3). Laterally, the dentary bears a longitudinal groove parallel to the oral margin of the bone. The surangular is slightly more than one third of the whole length of the mandible. The bone tapers anteriorly and overlies the elongate angular and the posterior process of the dentary. Posteriorly, the surangular forms the lateral wall of the articular fossa for the quadrate and abuts against the stout articular. The retroarticular process of the articular is relatively short, ending in a rounded posterior margin. The dorsal surface of the process has two large fossae, separated by a low and longitudinal ridge; the lateral fossa is deeper than the medial one (Fig. 3). By contrast, a longitudinal ridge is present on the dorsal surfaces of the retroarticular processes in *Wumengosaurus*, *Qianxisaurus* and *Dawazisaurus*, and a single fossa or trough in other pachypleurosaurs.

There are eight teeth preserved in each premaxilla. Considering two obvious gaps for missing teeth, the complete number would be ten. 12 teeth are discernable in left maxilla, and five or six teeth are missing (Fig. 3). The tooth number in the dentary is hard to estimate due to occlusion of jaws. The teeth are homodont with a tall peduncle and a short, conical crown. The lateral surface of the crown is nearly smooth.

**Figure 4:**
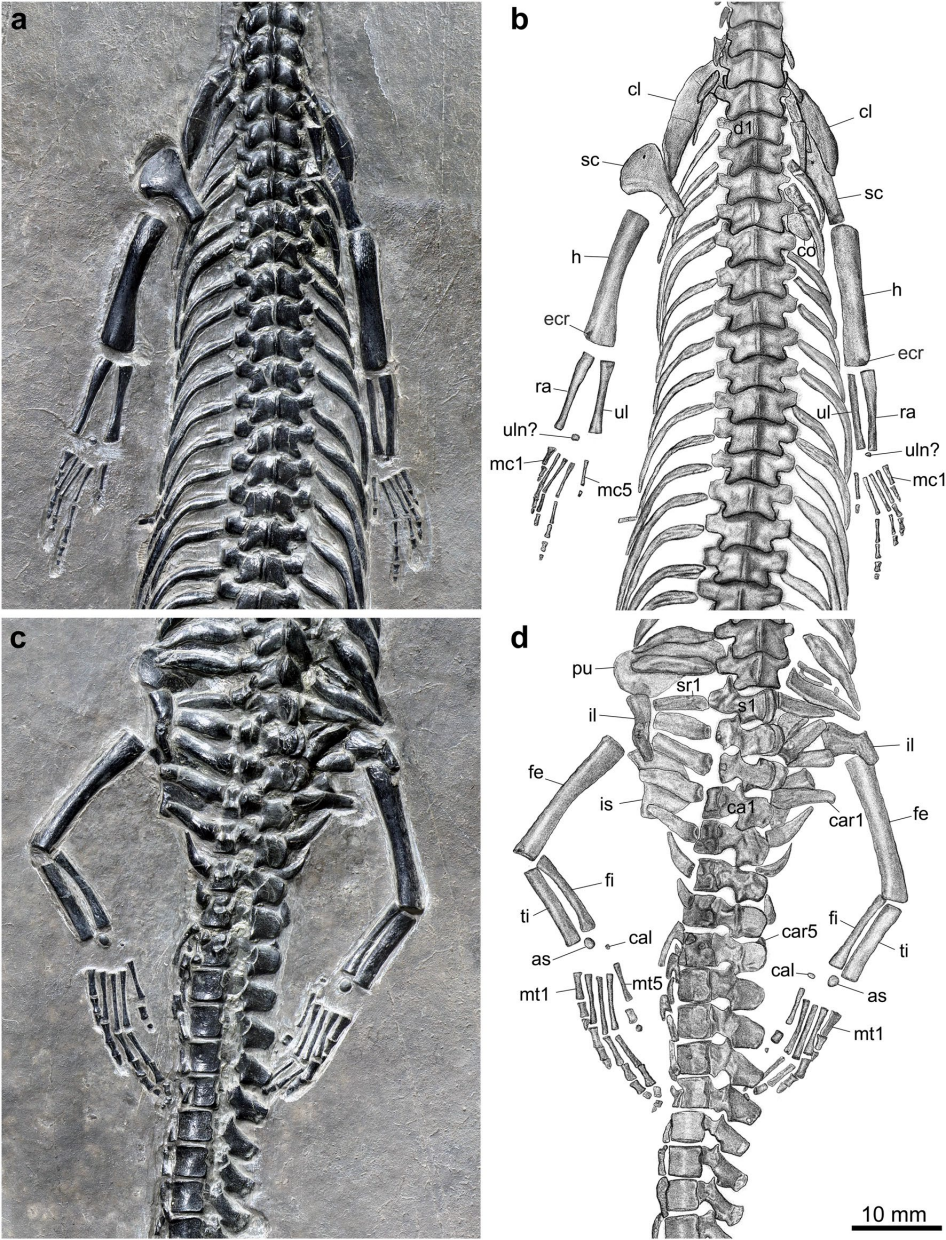
Girdles, limbs and vertebrae of *Honghesaurus longicaudalis* gen. et sp. nov., IVPP V30380. Photo (**a**) and line-drawing (**b**) of pectoral girdle, forelimbs and anterior dorsal vertebrae. Photo (**c**) and line-drawing (**d**) of pelvic girdle, hind limbs and posterior vertebrae. as, astragalus; ca, caudal vertebra; cal, calcaneum; car, caudal rib; cl, clavicle; co, coracoid; d, dorsal vertebra; ecr, ectepicondylar ridge; fe, femur; fi, fibula; h, humerus; il, ilium; is, ischium; mc, metacarpal; mt, metatarsal; pu, pubis; ra, radius; s, sacral vertebra; sc, scapula; sr, sacral rib; ti, tibia; ul, ulna; uln, ulnare.

The vertebral column consists of 121 vertebrae, including 20 cervical, 29 dorsal, three sacral, and 69 caudal vertebrae (Figs. 2, 4). The proatlas is small and triangular, and the atlas is presented by a pair of larger, trapezoid neural arches. The axis has a neural spine 1.3 times longer than that in the third cervical (Fig. 3). The cervicals show pachyostotic neural arches with a low neural spine. Some cervical ribs are discernable in the left side and their lengths increase posteriorly. The 21th vertebra is considered as the first dorsal vertebra because its rib is much longer than that of the last cervical rib (Fig. 4a). The dorsal ribs bow posteromedially and show distinct pachyostosis in posterior ones. The sacral ribs are relatively short and stout with the last being the longest. Five pairs of caudal ribs are present (Fig. 4c), similar to those in *Wumengosaurus* (three to five pairs) in number; by contrast, other pachypleurosauroids generally have seven or more pairs of caudal ribs. The first haemapophysis occurs in the fifth caudal, and the chevron bones continue up to the 23th caudal.

The exposed portion of the clavicle is broad and blade-like. It tapers posterolaterally and lacks an anterolaterally expanded corner (Fig. 4a). The interclavicle is unexposed. The scapula consists of a flat ventral portion and a relatively narrow dorsal wing. The width of the dorsal wing varies little through its length. The humerus is bowed posteromedially. The deltopectoral crest is hardly differentiated. A low ectepicondylar ridge is clearly present, and an entepicondylar foramen is absent in the expanded distal portion of the bone (Fig. 4a). The radius, slightly longer than the ulna (Table 1), is more expanded proximally than distally. The ulna is straight with a slightly constricted shaft. In each forelimb, there is a single, small and rounded carpal ossification, which likely represents the ulnare deduced from its shape. The intermedium is probably unossified. Among five metacarpals, Metacarpal I is the shortest with an expanded proximal end; Metacarpal II is longer than Metacarpal V but slightly shorter than Metacarpal IV (Table 1). The phalangeal formula is 2-3-2-4-1 for the manus.

In the pelvic girdle, only the iliums are well exposed, and they are roughly trapezoidal with a dorsal process (Fig. 4c). The plate-like pubes and ischiums are partially covered by the ribs and vertebrae, and no significant details are visible. The femur is slightly longer than the humerus with the proximal end slightly more expanded than the distal end (Fig. 4c). The tibia and fibula are nearly equal in length. The tibia is straight, thicker than the slightly curved fibula. Two ossified tarsals, calcaneum and astragalus, are rounded; the former is tiny and the latter significantly larger. All metatarsals are well preserved, and the relative lengths between them show a similar pattern in metacarpals (Table 1). The phalangeal count is 2-3-4-5-2 for the pes.

## Discussion

**Figure 5:**
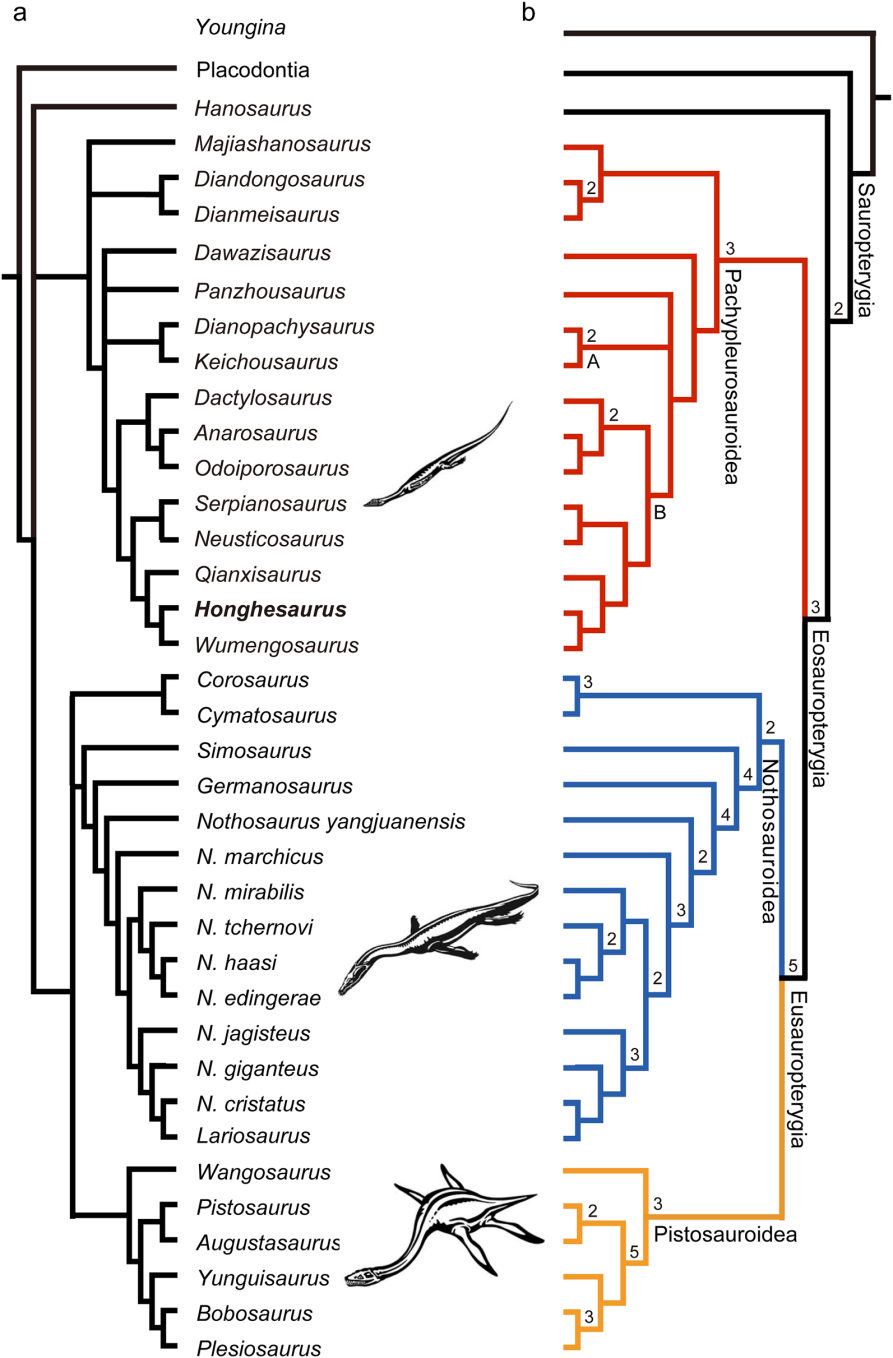
Phylogenetic position of *Honghesaurus longicaudalis* gen. et sp. nov. (**a**) Strict consensus of five trees rooted with four placodonts (TL = 686, CI = 0.3776, and RI = 0.6674). (**b**) Strict consensus of two trees rooted with *Youngina* (TL = 710, CI = 0.3662, and RI = 0.6649). Bremer decay indices larger than 1 are indicated above or below the nodes in tree a. Node A, Keichousauridae. Node B, Pachypleurosauridae.

To assess the phylogenetic affinities of *Honghesaurus*, we incorporated it into a dataset revised from a previous study^31^ (see “Methods” and Supplementary Information). Results of our phylogenetic analyses unite the pachypleurosauroids (excluding *Hanosaurus*) as a monophyletic group sister to the Eusauropterygia within the Eosauropterygia, largely consistent with refs.^15–17,20,28,31,33^ (but see refs.^19,24,27^). *Honghesaurus* is consistently recovered as a sister taxon of *Wumengosaurus* within the Pachypleurosauridae in two analyses rooted by four placodonts and the basal diapsid *Youngina capensis*, respectively (Fig. 5). However, the phylogenetic positions of *Majiashanosaurus* and *Panzhousaurus* are unstable. In the better resolved topology (Fig. 5b), *Majiashanosaurus* is united with *Dingdongosaurus* and *Dianmeisaurus* as a basal clade of Pachypleurosauroidea. Above *Dawazisaurus*, an unresolved trichotomy involves *Panzhousaurus*, Keichousauridae (*Keichousaurus* and *Dianopachysaurus*) and Pachypleurosauridae. Within the Pachypleurosauridae, three Chinese genera (*Qianxisaurus*, *Honghesaurus* and *Wumengosaurus*) consist of a monophyletic group which is more closely related the European clade *Neusticosaurus*-*Serpianosaurus* than to the other European clade *Dactylosaurus-Anarosaurus-Odoiporosaurus*. This suggests that the European pachypleurosaurids would be paraphyletic, and the three Chinese pachypleurosaurids (*Qianxisaurus*, *Honghesaurus* and *Wumengosaurus*) are likely direct descendants of European relatives.

*Honghesaurus* is referred to the Pachypleurosauridae as it shares the following synapomorphies of this family: the preorbital region distinctly longer than the postorbital region, presence of paired parietals, a pineal foramen close to the middle of skull table, more than 20 dorsal vertebrae (reversal in *Dactylosaurus*), and a concave anterior (preaxial) margin of the shaft of the radius (independently evolved in *Diandongosaurus*). The monophyly of the Chinese pachypleurosaurid clade *Qianxisaurus*-*Honghesaurus*-*Wumengosaurus* is well supported by presence of an elongated snout, a retracted external naris, a dorsal wing of the scapula varying little through its length (independently evolved in *Majiashanosaurus*, *Dingdongosaurus* and *Dianmeisaurus*), and absence of a trough on dorsal surface of retroarticular process (independently evolved in *Dawazisaurus*, and reversal in *Honghesaurus*). Within this clade, *Honghesaurus* is sister to *Wumengosaurus*, as both share several derived features, e.g., presence of an elongated snout with an anteriorly tapering rostrum, a jugal/squamosal contact, and a squamosal broadly separated from the ventral margin of the skull. The close affinities between *Qianxisaurus* and *Wumengosaurus* have been recognized in a previous study^31^, but there is a distinct morphological gap between them, and the new discovery of *Honghesaurus* narrows this morphological gap.

The discovery of *Honghesaurus* provides an importation addition for our understanding of the morphological diversity, ecological adaption and aquatic locomotion of pachypleurosaurs. *Honghesaurus* is easily distinguished from the closely related *Wumengosaurus* and other pachypleurosaurs by a series of autapomorphies (see Diagnosis and Description above). Notably, the presence of two fossae in the retroarticular process of *Honghesaurus* is unique in pachypleurosaurids. The medial fossa may receive the posteriorly projecting head of the quadrate, as previously suggested for the fossa in the retroarticular process of European pachypleurosaurids^14,15^. The lateral fossa, unknown in other pachypleurosaurids, is probably associated with a well-developed depressor muscle^38^. As typically in other pachypleurosaurids, the quadrate of *Honghesaurus* is deeply excavated posteriorly, indicating the presence of a relatively large tympanic membrane; this air-filled middle ear would restrict it to shallow marine environments^13–17^. The relatively slender humerus and long tail suggest that *Honghesaurus* would have relied mainly on lateral undulation of the trunk and tail for aquatic propulsion^13,18^. It is worthy to note that the postcranial axial skeleton of *Honghesaurus*, comprised of 121 vertebrae, is proportionally longest among pachypleurosaurs. The long trunk of *Honghesaurus* includes 29 dorsal vertebrae. The most comparable count (28 dorsal vertebrae) is present in *Wumengosaurus* and *Qianxisaurus*; by contrast, other pachypleurosaurs generally have 25 or less dorsal vertebrae. In addition, *Honghesaurus* has 69 caudal vertebrae, bearing the longest tail among pachypleurosaurs; other pachypleurosaurs commonly have no more than 58 caudal vertebrae (Table 2). The caudal ribs are reduced in number (five pairs), making that most of the tail is laterally compressed in *Honghesaurus*. Such a long trunk associated with an incredibly long tail could provide *Honghesaurus* the advantage of maneuverability and energy efficiency for lateral undulatory swimming^51^.

**Table 2:** Meristics for presacral, cervical and caudal vertebrae in pachypleurosaurs with relatively complete skeletons.

Taxon	Presacral	Cervical	Caudal	CP ratio	Reference
*Diandongosaurus acutidentatus*	38	19	40	0.50	Shang et al.^26^
*Dianmeisaurus gracilis*	45	23	41	0.51	Shang and Li^27^
*Panzhousaurus rotundirostris*	44	24	22+	0.54	Jiang et al.^22^
*Dawazisaurus brevis*	36	20	37	0.56	Cheng et al.^29^
*Dianopachysaurus dingi*	39	20	17+	0.51	Liu et al.^20^
*Keichousaurus hui*	44	25 or 26	37	0.58	Lin and Rieppel^18^
*Anarosaurus pumilio*	45	19 or 20	–	0.43	Rieppel and Lin^17^
*Dactylosaurus gracilis*	36	17	–	0.47	Rieppel and Lin^17^
*Serpianosaurus mirigiolensis*	35 to 38	15 to 18	58	0.45	Rieppel^15^
*Neusticosaurus pusillus*	41 to 43	18 to 20	51 to 58	0.45	Sander^14^
*Neusticosaurus peyeri*	35 or 36	15 or 16	42 to 48	0.44	Sander^14^
*Qianxisaurus chajiangensis*	46	18	31+	0.39	Cheng et al.^30^
*Wumengosaurus delicatomandibularis*	49	21	36+	0.43	Wu et al.^25^
*Honghesaurus longicaudalis*	49	20	69	0.41	This study

The CP ratio means the ratio between the number of cervical vertebrae to all presacral vertebrae.

The discovery of *Honghesaurus* documents the first really long-tailed pachypleurosaur, providing evidence for the vertebral multiplication in early sauropterygians. Inspired by previous studies on the evolution of vertebral numbers of amniotes^52,53^, we conducted a survey of presacral and caudal vertebral counts across all pachypleurosaurs represented by relatively complete skeletons (Table 2), and found that the ratios between the number of cervical vertebrae to all presacral vertebrae (CP ratio) in Pachypleurosauridae (CP = 0.39–0.47) are smaller than those in Keichousauridae and basal pachypleurosauroids (CP = 0.50–0.58), and that Pachypleurosauridae have 42 to 69 caudal vertebrae, contrasting no more than 41 caudal vertebrae in Keichousauridae and basal pachypleurosauroids (although the number in several pachypleurosaurs is unknown or incomplete because of poor state of preservation). The lower CP ratios (0.39–0.43) with highest numbers of presacral vertebrae (46–49) among pachypleurosauroids indicate both meristic and homeotic changes^52^ in the Chinese pachypleurosaurid clade *Qianxisaurus*-*Honghesaurus*-*Wumengosaurus*. The reduction of the CP ratio is clearly associated with the increase of the trunk length relative to the neck length, and the multiplication of caudal vertebrae increases the tail length. As such, there is an evolutionary trend towards the increase of trunk and tail lengths in derived pachypleurosaurs, which could functionally increase the energy efficiency for the lateral undulatory swimming in this clade^51^. This evolutionary trend has not been observed in plesiosaurs and other sauropterygians that were adapted for appendicular swimming^54,55^.

The discovery of *Honghesaurus* documents the first evidence of pachypleurosaurids from the Anisian Luoping Biota in Yunnan Province, providing new insights into the Middle Triassic radiation and palaeobiogeography of this group. Other pachypleurosaurs known from this biota are a keichousaurid^20^ and more plesiomorphic forms^26–29^. Stratigraphically, *Honghesaurus* is slightly older than *Wumengosaurus*, representing the oldest pachypleurosaurid in China. Even older pachypleurosaurids are known from Europe. *Dactylosaurus* from the earliest Anisian Lower Gogolin beds in the eastern Germanic Basin documents the oldest pachypleurosaurid in the world^12^. *Anarosaurus* from the late early Anisian Lower Muschelkalk Vossenveld Formation in the western Germanic Basin, is slightly younger than *Dactylosaurus* but is older than *Odoiporosaurus* and *Serpianosaurus* near the Anisian/Ladinian boundary in the Monte San Giorgio area, and the Ladinian *Neusticosaurus* represents the youngest pachypleurosaurid in the Germanic and Alpine Triassic^13–17,19^. Based on these stratigraphic data combined with the scenario of pachypleurosaurian phylogeny newly proposed here, we suggest that the Pachypleurosauridae may originate in the western Tethys as early as the earliest Anisian, and from there, this family diversified and dispersed into the eastern Tethys at the middle Anisian. However, the superfamily Pachypleurosauroidea may originate in the eastern Tethys based on the occurrence of the earliest and basal taxa in that realm^21^. The unnamed pachypleurosaur recently recovered from Myanmar^39^ potentially represents one of the oldest pachypleurosauroids and corroborates an eastern Tethyan origin for this clade, but it apparently needs further studies. The biogeographic evolution of pachypleurosauroids is probably more complicated than the previously thought^35^.

## Methods

### Phylogenetic analysis

In order to assess the phylogenetic position of *Honghesaurus* within the Eosauropterygia, we incorporated it into a matrix slightly expanded from that of ref.^31^. We added a new character (Char. 149), giving a total of 149 characters coded across 51 taxa in the current data matrix (Supplementary Material). Besides *Honghesaurus* described here, *Dawazisaurus* was added as well, so all pachypleurosaurs based on well-preserved material were included in our data matrix. *Lariosaurus sanxiaensis* was not included due to absence of any cranial information. We first used four well-studied placodonts (*Placodus*, *Paraplacodus*, *Cymodus* and *Psephoderma*) for out-group comparison, following ref.^31^. However, several nodes are not well resolved in this analysis. For better analyzing the character polarity, we further added the basal diapsid *Youngina capensis *to root the tree in the second analysis. The data matrix was generated by WinClada (v. 1.00.08)^56^. The maximum parsimony analyses were performed with a heuristic search in PAUP* (v. 4.0a169)^57^ using 500 random addition sequence replicates, holding five trees at each step, with the tree bisection and reconnection (TBR) strategy enabled and maxtrees set to automatically increase by 100.

### Nomenclatural acts

The nomenclatural acts for the new genus and species have been registered in the proposed online registration system (ZooBank) for the International Code of Zoological Nomenclature (http://zoobank.org/). The Life Sciences Identifier for this paper is urn:lsid:zoobank.org:pub:3FD01E12-25B0-4C22-8868-09B36DAA61DA.

## Supplementary Information

Supplementary Information.

## Data Availability

The data that support the findings of this study are available in the Supplementary Information.
