# Supplementary material for: A long-tailed marine reptile from China provides new insights into the Middle Triassic pachypleurosaur radiation
Source: Sci Rep. 2022 May 5;12:7396. doi: 10.1038/s41598-022-11309-2 (PMC9072359; doi:10.1038/s41598-022-11309-2)
Supplement: Supplementary file 1 — Supplementary Information. [file 41598_2022_11309_MOESM1_ESM.pdf]

Supplementary materials for

A long-tailed marine reptile from China provides new insights into the Middle Triassic pachypleurosaur radiation

Guang-Hui Xu[1,2], Yi Ren[1,2,3], Li-Jun Zhao[4], Jun-Ling Liao[5] & Dong-Hao Feng[1,2,3]

[1]Key Laboratory of Vertebrate Evolution and Human Origins of Chinese Academy of Sciences, Institute of Vertebrate Paleontology and Paleoanthropology, Chinese Academy of Sciences, Beijing 100044, China

[2]CAS Center for Excellence in Life and Paleoenvironment, Beijing 100044, China

[3]University of Chinese Academy of Sciences, Beijing 100049, China

[4]Zhejiang Museum of Natural History, Hangzhou 310014, China

[5]College of Resource and Environmental Engineering, Guizhou University, Guiyang 550025, China

Part A. Supplementary figures

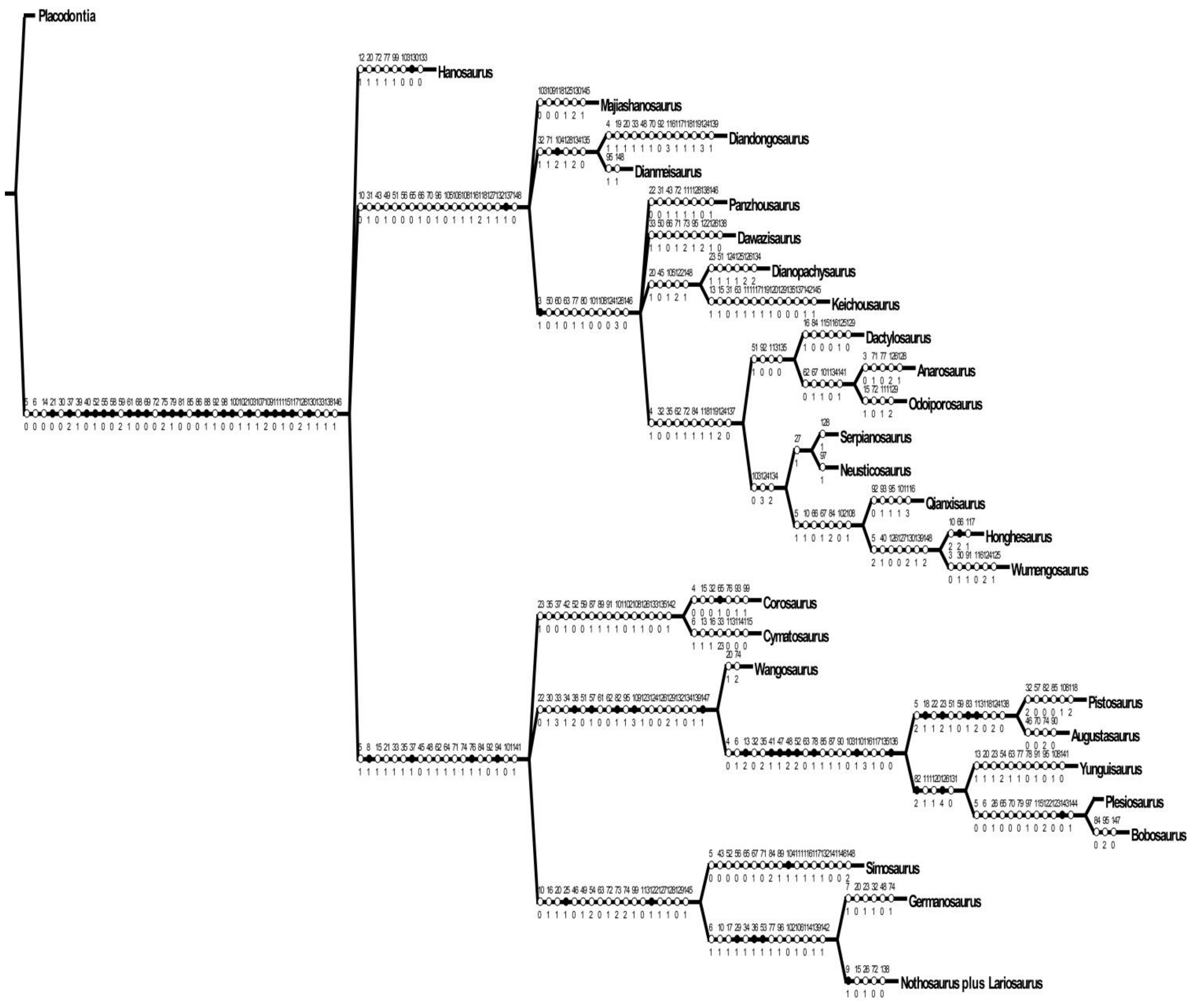

Figure 1S. Strict consensus of five most parsimonious trees rooted with four placodonts (TL = 686, CI = 0.3776, and RI = 0.6674), showing all character optimisations. The topologies within *Nothosaurus* and *Lariosaurus* are omitted.

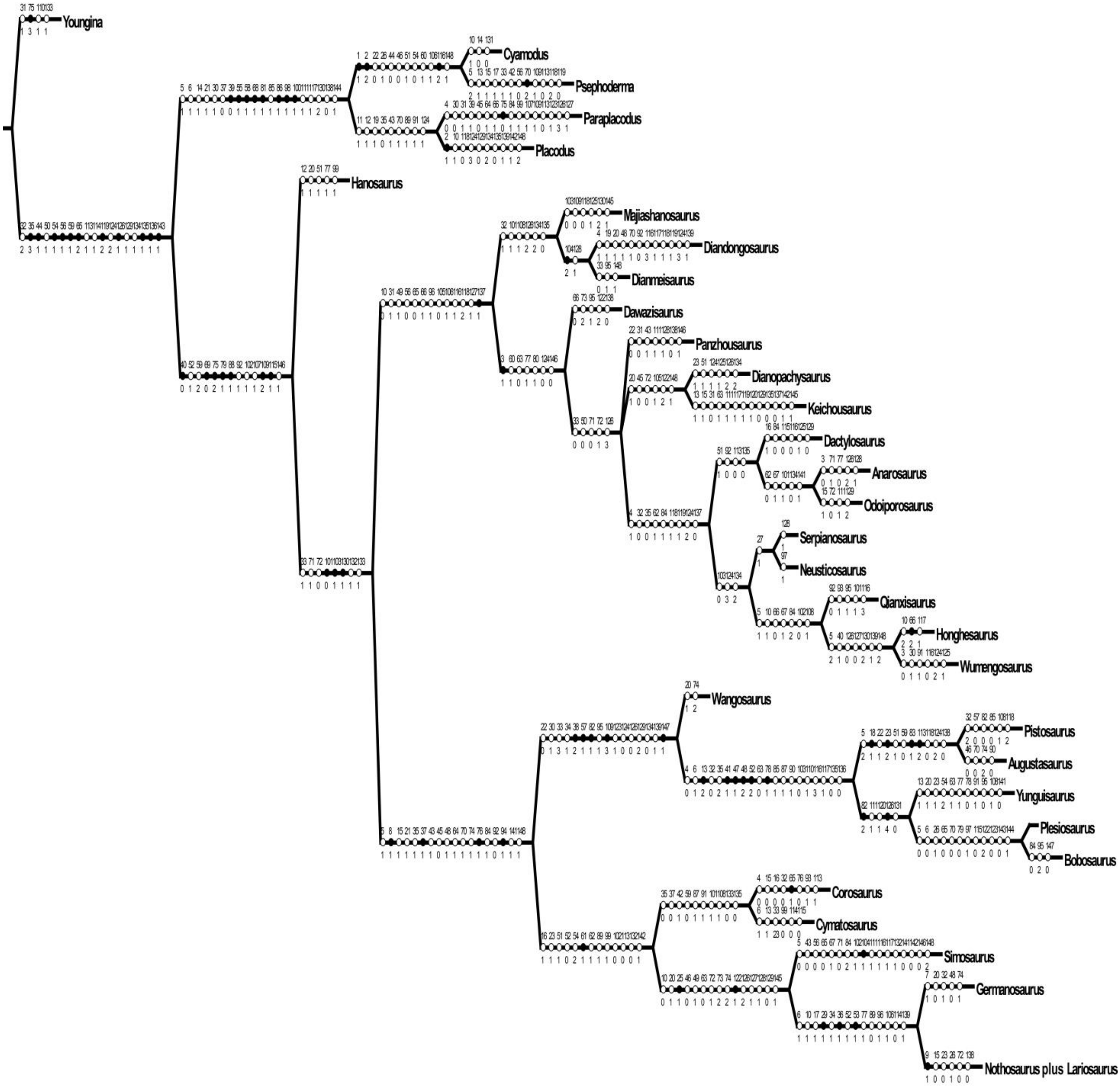

Figure 2S. Strict consensus of two most parsimonious trees rooted with *Youngina* (TL = 710, CI = 0.3662, and RI = 0.6649), showing all character optimisations. The topologies within *Nothosaurus* and *Lariosaurus* are omitted.

Part B. Character list

We incorporated *Honghesaurus longicaudalis* gen. et sp. nov. into the data matrix of Lin et al. (2021), which in turn based mainly on the data matrixes of Rieppel et al. (2002), Rieppel and Lin (1995), Benson et al. (2012), Li et al. (2014), Liu et al. (2014), Neenan et al. (2015), Ma et al. (2015), Lin et al. (2019), and Li and Liu (2020). Following Lin et al. (2021), original character sequences of Rieppel et al. (2002) are indicated in the end of each character description with an R before the number.

(1) Body contours (trunk): elongated (0); broad and rounded (1). (From Li et al., 2014:144)
(2) Dermal armour ('osteoderms'): absent (0); present (1); forming carapace (2). (From Neenan et al., 2015: 138; Lin et al., 2021)
(3) Bones in dermatocranium: distinctly sculptured (0); relatively smooth (1). (From Rieppel and Lin, 1995:1)
(4) Preorbital and postorbital region of skull: of subequal length (0); preorbital region distinctly longer (1); postorbital region distinctly longer (2). (R12)
(5) Snout: relatively short (0); elongated with broad anterior termination (1); elongated and tapering anteriorly (2). (R132)
(6) Distinct snout constriction in adult: absent (0); present (1). (R3)
(7) Premaxilla(e) in adult: paired (0); partly or fully fused (1). (From Liu et al., 2014:7)
(8) Ascending process of maxilla: distinct (0); reduced (low) (1). (From Li et al., 2014:138)
(9) Maxilla, depression at lateral margin of external naris and a foramen at its bottom for the exit of a lateral branch of superior alveolar nerve: absent (0); present (1). (From Liu et al., 2014:8)
(10) External nares: not retracted (0); retracted with a longitudinal diameter approaching or exceeding half the longitudinal diameter of orbit (1); retracted, narrow, and with a longitudinal diameter distinctly less than half the longitudinal diameter of orbit (2). (R133)
Remarks: The coding for *Dianmeisaurus* is changed from '2' to '0' based on Shang et al. (2017).
(11) Nasals in adult: paired (0); fused (1). (From Liu et al., 2014:9)
(12) Nasal(s): shorter than frontal(s) (0); longer than frontal(s) (1). (R5)
(13) Nasal(s): not reduced (0); reduced (1); absent (2). (R6)
(14) Nasal, anterolateral process lining the entire medial margin of external naris: absent (0); present (1). (From Liu et al., 2014:11)
(15) Nasal(s): meeting each other (0); separated from one another by nasal processes of the premaxillae extending back to frontal bone(s) (1). (From Liu et al., 2014:10)

(16) Nasal-prefrontal contact: present (0); absent (1). (From Liu et al., 2014:14)
(17) Dorsal exposure of prefrontal: large (0); reduced (1). (R11)
(18) Prefrontal: without slender anteromedial process (0); with slender anteromedial process entering between maxilla and premaxilla (1). (R121)
(19) Frontal: participating in the formation of dorsal margin of orbit (0); excluded from dorsal margin of orbit by a contact of prefrontal and postfrontal (1). (R10)
(20) Frontal(s) in adult: paired (0); fused (1). (R14)
(21) Lateral edge of frontal: concave (0); rather straight or slightly convex (1). (From Rieppel and Lin, 1995)
(22) Distinct posterolateral processes of frontal(s): absent (0); present (1). (R15)
(23) Frontal: widely separated from upper temporal fossa (0); narrowly approaching upper temporal fossa (1); entering the anteromedial margin of upper temporal fossa (2). (R16)
Remarks: The codings for *Diandongosaurus, Dianmeisaurus* and *Odoiporosaurus* are changed from ‘1’ to ‘0’ based on Shang et al. (2011), Shang et al. (2017) and Renesto et al. (2014), respectively.
(24) Postfrontal: with distinct lateral process overlapping the dorsal tip of postorbital (0); with reduced lateral process and hence more of an elongate shape (1). (R26; Lin et al., 2021).
(25) Postfrontal, distinct constriction behind the orbit: absent (0); present (1). (From Liu et al., 2014: 25)
(26) Postfrontal: entering upper temporal fossa (0); excluded from upper temporal fossa (1). (From Liu et al., 2014:26)
(27) Postorbital: in the formation of anterior margin of upper temporal fossa (0); excluded from anterior margin of upper temporal fossa by a contact of postfrontal and squamosal, or entering it narrowly (1). (From Lin et al., 2019:27)
Remarks: The coding for *Serpianosaurus* is changed from ‘0’ to ‘1’ based on Rieppel (1989).
(28) Jugal: present (0); absent (1). (From Liu et al., 2014:21)
(29) Jugal: entering orbit (0); excluded from posterior margin of orbit (1). (From Liu et al., 2014:22)
(30) Jugal: excluded from upper temporal arch (0); entering upper temporal arch (1). (R25)
(31) Distinctly open L-shaped (boomerang-shaped) jugal: absent (0); present (1). (From Neenan et al., 2015: 139)
(32) Parietal(s) in adult: paired (0); fused in their posterior part only (1); fully fused (2). (R17)
(33) Parietal skull table: broad (0); weakly constricted (1); strongly constricted (at least posteriorly) (2); forming a sagittal crest (3). (R19)

(34) Parietal skull table, constriction in the posteriormost part: absent (0); present (1). (From Liu et al., 2014:19)
(35) Pineal foramen: close to the middle of skull table (0); weakly displaced posteriorly (1); strongly displaced posteriorly (2); displaced anteriorly (3). (R18; Lin et al., 2021)
Remarks: The coding for *Qianxisaurus* is changed from ‘3’ to ‘0’ based on Cheng et al. (2012).
(36) Temporal region of skull: relatively high (0); strongly depressed (1). (R4)
(37) Ratio of longitudinal diameters, upper temporal fossa to orbit: between 1.0 and 2.0 (0); 2.0 or more (1); less than 1.0 (2). (Modified from Liu et al., 2014)
(38) The anteromedial corner of upper temporal fossa: not (0); partially (1); fully floored by a descensus from postorbital, which together with neighbouring elements (postfrontal, parietal) separates it from orbit (2). (R122)
(39) Lower temporal fossa: absent (0); present (1). (R27; Lin et al., 2021)
(40) Squamosal: descending to ventral margin of skull (0); broadly separated from ventral margin of skull (1). (R28)
Remarks: The codings for *Keichousaurus* and *Qianxisaurus* are changed from ‘1’ to ‘0’ based on Lin & Rieppel (1995) and Cheng et al. (2012), respectively.
(41) A box-like suspensorium of squamosal: absent (0); present (1). (R123)
(42) Distinct notch of squamosal to receive distal tip of paroccipital process: absent (0); present (1). (R32)
(43) Quadratojugal: present (0); absent (1). (R29)
(44) Anterior process of quadratojugal: present (0); absent (1). (R30)
(45) Posterior margin of quadrate: straight (0); concave (1). (R37)
(46) Dorsal wing of epipterygoid: approximately as broad as its base (0); narrower than its base (1). (R39)
(47) Braincase: located at posterior end (0); deeply recessed below parietal skull roof (or parietal sagittal crest) (1). (R124)
(48) Occipital crest: absent (0); present but squamosals not meeting behind parietal (1); present and squamosals meeting behind parietal (2). (R36)
(49) Occiput: paroccipital processes trending posteriorly (0); plate-like with no distinct paroccipital process and with strongly reduced posttemporal fossae (1). (R31; Lin et al., 2021)
(50) Occiput (posterior margin of skull table): nearly straight (0); deeply concave (excavated) (1). (From Li et al., 2014:139)
(51) Mandibular articulations: approximately at level with occipital condyle (0); displaced to a level distinctly behind occipital condyle (1) (R33; Lin et al., 2021)
(52) Supraoccipital: exposed more or less vertically on occiput (0); exposed more or less horizontally at posterior end of parietal skull table (1); U-shaped (2). (R35)

(53) Supraoccipital: below the occipital exposure of the parietal (0); sutured with the parietal horizontally (1). (From Ma et al., 2015:141)
(54) Basioccipital tubera: free (0); in complex relation to pterygoid, as they extend ventrally (1); in complex relation to pterygoid, as they extend laterally (2). (R42)
(55) Vomer contact pterygoid: present (0); absent (1). (From Li et al., 2014:146)
(56) Premaxillae: entering internal naris (0); excluded from internal naris (1). (R45)
(57) Posterior palatine vacuities (Andrews, 1896; distinct medial emargination [concavity] on quadrate ramus of pterygoid behind palatobasal articulation): absent (0); present (1). (R125)
(58) Pterygoids: longer than palatines (0); shorter than palatines (1). (R130)
(59) Pterygoid flanges: well developed and transversely oriented (0); well developed and longitudinally oriented (1); strongly reduced (2). (R44)
(60) Ectopterygoid: present (0); absent (1). (R46)
(61) Internal carotid passage: entering basicranium (0); entering quadrate ramus of pterygoid (1). (R47)
(62) Splenial bone: entering mandibular symphysis (0); excluded there from (1). (R52)
(63) Distinct coronoid process of lower jaw: absent (0); present (1). (R49)
(64) Strongly projecting lateral ridge of surangular defining the insertion area for superficial adductor muscle fibres on the lateral surface of lower jaw: absent (0); present (1). (R50)
(65) Mandibular symphysis: short (0); somewhat enforced (1); elongated and ‘scoop-like’ (2). (R51)
(66) Trough(s) on dorsal surface of retroarticular process: absent (0); single trough (1); two troughs (2). (Modified form Rieppel and Lin, 1995:23)
Remarks: The character state (2) has been added, for coding the condition in *Honghesaurus* described here.
(67) Marginal teeth, lingual surface of crown: convex (0); concave (1). (From Li et al., 2014:149)
(68) Durophagous dentition: absent (0); present (1). (R128)
(69) Number of premaxillary teeth: four or more (0); three or less (1). (R129)
(70) Anterior (premaxillary and dentary) teeth: upright or only slightly procumbent (0); strongly procumbent (1); absent (2). (R54; Neenan et al., 2015)
(71) Premaxillary and anterior dentary fangs: absent (0); present (1). (R55)
(72) One or two enlarged teeth on maxilla: present (0); absent (1). (R56)
(73) Number of small maxillary teeth anterior to the maxillary fang(s): 3 or less (0); 4 (1); 5 or more (2). (Modified from Liu et al., 2014:46)
(74) Maxillary tooth row: restricted to a level in front of the posterior margin of orbit (0); extending backwards to a level below the posterior corner of orbit and/or the

anterior corner of upper temporal fossa (1); extending backwards to a level below the anterior one third to one half of upper temporal fossa (2). (R57)
(75) Palatine dentition: single row with four or more teeth (0); single row with three to one teeth/tooth (1); absent (2); multiple rows with small numerous teeth/denticles (3). (From Neenan et al., 2015:140)
(76) Vertebrae: amphicoelous (0); platycoelous (1); procoelous/opisthocoelous (2). (R60)
(77) Vertebral centrum: distinctly constricted in ventral view (0); with parallel lateral edges (1). (R67)
(78) Subcentral foramina: absent (0); present (1). (R127)
(79) Zygosphene-zygantrum articulation: absent (0); present (1). (R64)
(80) Zygapophyseal pachyostosis: absent (0); present (1). (R69)
Remarks: The coding for *Wumengosaurus* is changed from ‘0’ to ‘1’ (Wu et al., 2011: fig. 4).
(81) Neural canal: evenly proportioned (0); distinctly higher than wide (1); wider than high (2). (From Li et al., 2014:140)
(82) Number of cervical vertebrae: 30 or below (0); more than 30 but less than 40 (1); 40 or more (2). (R134; Li et al. 2014)
(83) Parapophysis shifting backwards on centrum along cervical vertebral column: absent (0); present (1). (R135)
(84) Number of dorsal vertebrae: 20 or below (0); more than 20 but less than 28 (1); 28 or more (2). (Modified from Rieppel and Lin, 1995:30; Li and Liu, 2020:106)
Remarks: The character state (2) has been revised from ‘30 or more’ to ‘28 or more’, for distinguishing the conditions in *Wumengosaurus*, *Honghesaurus* and *Qianxisaurus* from those in other pachypleurosauroids.
(85) Distal articular surface on transverse processes of dorsal vertebrae: oblong (0); evenly rounded (1). (R136)
(86) Transverse processes of neural arches of the dorsal region: relatively short (0); distinctly elongated (1). (R66)
(87) Distal end of transverse processes of dorsal vertebrae: not increasing in diameter (0); distinctly thickened (1). (R68)
(88) Sutural facets receiving pedicels of neural arch on dorsal surface of centrum in dorsal region: narrow (0); expanded into a cruciform or ‘butterfly-shaped’ platform (1). (R65)
(89) Anteroposterior trend of increasing inclination of pre- and postzygapophyses within dorsal and sacral region: absent (0); present (1). (R70)
(90) Neural spines on dorsal vertebrae: low (0); tall (1). (From Liu et al., 2014: 48)
(91) Elongation of neural spines in proximal tail region: (0) absent; (1) present, spines as high as the corresponding vertebral length or higher. (Modified from Liu et al.,

2014:49)
(92) Pachyostosis of dorsal ribs: absent (0); present (1). (R72)
(93) Distinct groove on the posterior aspect of the proximal shoulder region of the dorsal ribs: absent (0); present (1). (R120)
(94) Last dorsal rib: longer than first sacral rib (0); shorter than first sacral rib (1). (From Lin et al., 2019:98)
(95) Number of sacral ribs: three or less (0); four (1); five or more (2). (R73; Lin et al., 2017)
(96) Distinct expansion of distal head of sacral ribs: present (0); absent (1). (R74)
(97) Number of segments included in each gastral rib: five or more (0); three (1). (Modified from Rieppel and Lin, 1995:34)
(98) Median gastral element: angulated (0); straight (1). (R131)
(99) The medial gastral rib element: with a single lateral process (0); May with two-pronged lateral process (1). (R119)
(100) Lateral gastral rib element: straight laterally (0); bent upwards laterally (1). (From Li et al., 2014:143)
(101) Clavicles: broad medially (0); narrow medially (1). (R77)
(102) Clavicles: not meeting in front of interclavicle (0); meeting in an interdigitating anteromedial suture (1). (R79)
(103) Anterolaterally expanded corners of clavicles: absent (0); present (1). (R80)
Remarks: The codings for *Qianxisaurus* and *Wumengosaurus* are changed from '1' to '0' based on Cheng et al. (2012) and Wu et al. (2011), respectively.
(104) Clavicle, anteriolateral process: absent (0); present, more medial (1); present, more lateral (2). (From Lin et al., 2019:109)
(105) Interclavicle: rho0mboidal (0); T-shaped (1); triangular (2). (R82; Liu et al., 2014).
(106) Posterior process on (T-shaped) interclavicle: elongate or relatively short (0); rudimentary or absent (1). (R83)
(107) Scapula: represented by a broad blade of bone (0); with a constriction separating a ventral glenoidal portion from a posteriorly directed dorsal wing (1). (R84)
(108) Dorsal wing or process of eosauropterygian scapula: tapers to a blunt tip (0); ventrally expanded at its posterior end (1). (R85)
(109) Coracoid: of rounded contours (0); slightly waisted (1); strongly waisted (2); with expanded medial symphysis and ridge-like thickening of the bone extending from glenoid facet posteriorly along lateral edge of the bone (3). (R88)
(110) Foot: short and broad (0); long and slender (1). (R112);
(111) Humerus: short than femur, or approximately of same length (0); longer than femur (1). (Modified from Lin et al., 2019:116)

(112) Humerus, proximal width compared with the mid-shaft: greater (0); less (1). (From Liu et al., 2014:58)
(113) Deltopectoral crest: well developed (0); reduced (1); absent (2). (R93)
(114) Insertional crest for latissimus dorsi muscle: prominent (0); reduced (1). (R94)
(115) Epicondyles of humerus: prominent (0); reduced (1). (R95)
(116) Ectepicondylar groove on humerus: open notched anterior (0); open without notched anterior (1); closed (i.e. ectepicondylar foramen present) (2); absent (3). (R96)
(117) Entepicondylar foramen: present (0); absent (1). (R97)
(118) Radius, anterior (preaxial) margin of shaft: smoothly curved (0); concave (1); rather straight (2). (Modified from Lin et al., 2019:124)
(119) Radius: shorter than ulna (0); longer than ulna (1); approximately of same length (2). (R98);
(120) Ulna, mid-diaphysis: slender (0); broadened (1). (From Liu et al., 2014:64)
(121) Ulna, anterior margin: smoothly concave (0); with a tuberosity (1). (From Liu et al., 2014:66)
(122) Ulna, posterior (postaxial) margin: concave (0); rather straight (1); convex (2). (From Lin et al., 2019:128)
(123) Distal end of ulna: not expanded, narrower than proximal part (0); distinctly expanded to at least the width of proximal part (1). (R126)
(124) Total number of carpal ossifications: five or more (0); four (1); three (2); one or two (3). (Modified from R137).
Remarks: The character state (3) has been modified form ‘two’ to ‘one or two’, for coding the condition in *Honghesaurus*.
(125) Intermedium: rounded (0); rectangular elongate (1). (From Rieppel and Lin, 1995:44)
(126) Iliac blade: well developed (0); reduced but projecting beyond level of posterior margin of acetabular portion of ilium (1); reduced and no longer projecting beyond posterior margin of acetabular portion of ilium (2); absent, (i.e., reduced to simple dorsal stub) (3). (R99; Neenan et al., 2015)
(127) Pubis: of rounded contours (0); waisted (1). (From Lin et al., 2019:133)
(128) Pubis: with convex ventral (medial) margin (0); with concave ventral (medial) margin (1). (R100; Li et al., 2014)
(129) Obturator foramen in adult: closed (0); open (1); absent (2). (R101; Li et al., 2014)
(130) Thyroid fenestra: absent (0); present (1); reduced (2). (R102; Li et al., 2014)
(131) Femoral shaft: stout and straight (0); slender and sigmoidally curved (1). (R104)
(132) Internal trochanter: well developed (0); reduced (1). (R105)
(133) Intertrochanteric fossa: distinct but reduced (0); rudimentary or absent (1).

(R106; Lin et al., 2021)
(134) Total number of tarsal ossifications: four or more (0); three (1); two or less (2). (R115)
(135) Proximal concavity of astragalus: absent (0); present (1). (R110)
(136) Distal tarsal 5: present (0); absent (1). (R114)
(137) Pes, ungual phalanges extremely expanded: absent (0); present (1). (From Lin et al., 2019:145)
(138) Nasal, length behind level of posterior margin of external naris more than twice of the maximal width: absent (0); present (1). (From Rieppel, 2001)
(139) Jugal-squamosal contact: absent (0); present (1). (From Liu et al., 2014)
Remarks: The coding for *Wumengosaurus* is changed from '0' to '1' based on Wu et al. (2011).
(140) Pineal foramen located within a deep trough: absent (0); present (1). (From Liu et al., 2014)
(141) Mandibular symphysis, anterior fusion: absent (0); present (1). (From Liu et al., 2014).
(142) Retroarticular process: short (0); long (1). (From Liu et al., 2014)
(143) Cervical vertebrae, proportions of anterior cervical neural spines: taller than their anteroposterior length (0); longer than tall (1). (Modified from Benson et al., 2012)
(144) Rib facets of the anterior-middle cervical vertebrae: separated (0); co-jointed (1). (Modified from Benson et al., 2012)
(145) Ulna, distinctly broadened proximal head compared with the radius: absent (0); present (1). (Modified from Rieppel, 2001)
(146) Hyperphalangy in manus: absent (0); present (1). (From Rieppel et al., 2003)
(147) Acetabular portion of ilium: broadened (0); rod-like (1). (From Li and Liu, 2020)
(148) Spina praeacetabuli: absent (0); weakly developed (1); well developed (2). (From Li and Liu, 2020)
(149) Little contribution of premaxilla to the medial border of the external naris: absent (0); present (1). (Character newly added here)

Part C. Data matrix

Youngina

0002000002000000000000100000000100000201100001?000000000000000?1000001
001?0300000000000000000000?0001000100?01000000011000000000000010100001
00000000000

Paraplacodus

00001?0002??01?00?1?1??00000001??0?000110?110?0???0???1?01????1121011101
?00000?010?11100?0100?0001111000??1?1010010?1120001103101210011?000??0?
?0?000

Placodus

0102110001110100001(01)11000000010(02)0000000100111100010001110110001020
0011101?01000001000110010100000010110001 00?00101100102000030100021002
010010011100020

Cyamodus

1202110001(01)0000000001(01)00010001020030000100001000011000110111 0?102
0011001?01000001000?1000000??00010(01)?000?10?0??0110??1200002?100120??
????000001?0?010

Psephoderma

12022100020011101000100001000102103000010100100001100?100111??????0112
01?0100??0?0???10?00000?000101??????0?10100102120000 0??1001210011100000
01?0?010

Hanosaurus

000200??0?010?000001010?000000?20?30201000????00?1110?????????????00??01
?0?010???????????0?1????00101?00????20????????????????0010100111??00?0????
??0

Majiashanosaurus

00???00
0?????0000????1?0010000110001110000 1?11002000021?10121????1????????1????

Anarosaurus

0001000000000000000000100000000100000201000??110010110?000021?00001100
011?020001100010001000000(01)100001110(01)11(01)2000011101100002021111 11
1001010010100?000

Dactylosaurus

0011000000000000100000100000000100000201000??110010110?000021?10001000
001?0201011000000001000 00?0100000110??1020?0010001100002131001?1?101010
0001000000

Serpianosaurus

001100000000000000000010000100010000020100001110010010?000021?10001000
001?02010110001000100 0(01)00(01)100000100011020(01)0(01)11101(12)00003031

1111112110100001000000

Neusticosaurus

00(01)10000000000(01)00000(01)1000010001000002010000111001001 0?000021?(
01)0001000001?0201011000100010001 00(01)110000100(01)11020(01)0(01)1(01)(0
1)0110000(23)(01)310(01)1111(12)110100001000000

Odoiporosaurus

00110000000000100000010000000010000020100001 1?00?01???????????00??10000
0?0?010110???000100000???0000??10????20100111011 0000??31021111????100?0?
?0?000

Honghesaurus

00112000020000000000001000000001000002011 00??1?0010010?????????00?2?000
01?0???0?1?002000?00010001??????00??11?00011111110000 30?????1??2?10110?0
100???1

Wumengosaurus

0001200001000000000000100000001100000201100011?00?00????0?021??00?01000
01?0201011?002000100110001000000000011120(01)0111001100002100012111211?1
10?01000020

Panzhousaurus

00120000000000000000000000000(12)003020100011 1?0010010???002???00?10
00001?020??1100000001000 1(01)0010000???0??1020101111022000000311111 11(0
1)111000?01001000

Dianopachysaurus

0012000000000000000101100000000120030201 00?0?0?00?01?0?????????00?10000
0000?????1?0?0?00?00010001?0000?10??10?000?11102200201121?1?1112111100?
01?0?010

Dawazisaurus

00120000000000000000100000000121030201000????00?1010?????????00?00000
1020????11?000?00?00010011??????10??10?000?1110220020001????1111111000?0
1?0?0?0

Diandongosaurus

000100000000000000110100000000111030201000011101 11010?000020?010010001
10002?0010?00000010000?00100001112011120001113111000030?11111112011?10
001001000

Dianmeisaurus

000200000000000000000100000000 1(12)0030201000??1?0011010?000020?010010
00010002?0010?000?00?000100110000111201112000111102200002021111111 2011
100001001010

Keichousaurus

001200000000101000010100000000002003020100001 0?0010010?000021?01001000

(01)00?020101100000001000100010000011011102010(01)(01)111211020003100111
11010100011010010
Qianxisaurus
001110000100000000000100000000100000201000011?0010010????????00?01000
01?0?010?1?002000?000010110000 1?00??112000?1130110000 3031?111112110100?
0100?001
Simosaurus
000200010000001(01)00011(01)(01)01000000210101010000100011110020000 2011
0100100101?2210010000 2000110000?0000100111101020100111112001020211 0111
1111010000??10020
Germanosaurus
000211110100001110001110100 01?01111110100???0?00?1??????0?????????000111
?12???110????????0
Nothosaurus yangjuanensis
000211011100000010011100110010021111101000110?0111111?010020???1?000011
0222110100001000100000(01)11100100010011?20000010012110010211011 0111100
10?11?11010
Nothosaurus marchicus
000211011100010110011100 1(01)001002211110100 0110?01100112010 0201?01200
0011022211011000000100?00?010010???0??1?20010010012001020211011 00111?
010111?1?010
Nothosaurus mirabilis
000211011100010110011101 0100(01)0022(01)11101000010001110112010 02011012
0000110122110110?0?000101000??100?00110111020100 0100??0010??21111100???
?11011????010
Nothosaurus giganteus
00021101110001(01)1100110010?0010021(01)11101000110?0111111?01000011012
000011012211011000100010 0000?210010101001102000101101210101021111 10011
10100111?10010
Nothosaurus tchernovi
0002110101000100 10011?010?01???22121101000??0?011001120?00201??12?00011
0122110110???000101?????????????????????000110?????????????1??????1????????
??0
Nothosaurus haasi
000111010000000111001110 00001???22121101000110001100112010020?????00011
0022???110???000??1?????????????????????100110????????????100????1?0??????
??0
Nothosaurus jagisteus
000211011100010110011000 01001002211110100 0?10?011001120?0000?101200001

1012211011000?000100000???0010001010102 0?0001101000002 0?111?????????001
1101???0
Nothosaurus edingerae
000211010100011?10011101000????22111101000??0?01100112010020??????00011
0022???0?1????????
0
Nothosaurus cristatus
00021101110001011001100001101002312110100 01?0001100112010000??????0001
1002???100???????
?0
Lariosaurus winkelhorsti
000211111210000110011000010010020001001000110001100 1?2????00??????00011
0012???110????????
0
Lariosaurus juvenilis
000211011200010010011100000010021011001000?100011011120100????????0001
?0022??100???????
?0
Lariosaurus youngi
00021101120000001001100001001002 2011(01)01000110?011001120?0000?(01)012
0000110(01)2211011000100010001011100(01)000(01)0(02)11?200(01)11100121100
003100110111101 01(01)11011000
Lariosaurus hongguoensis
000211011200010110011100010 1?00210110010 00110?011001?20?00?0?101200001
10022110???00???????0?????0000??0???1?200111110021 01000?10111112010?0101
1?11??0
Lariosaurus vosseveldensis
0002111112100100101111000100100211110010 00110?0110011??????????1?000001
0??000????????
0
Lariosaurus buzzii
000211111210000110011100000????21101001000??0?01100112010020?101200001
10(01)2211011000?000100000??100001?00??1120000111111 1010?031011111????1?
0011?1?000
Lariosaurus curionii
00021001120001 1?100111010101?0021111001000??0?011001120?0020?101200001
1002211011000?0001?0010???????011021102??111110101100????????????????0?11
01???0
Lariosaurus balsami

000211011000000?100111010?00000221(12)1001000??0?01100112010020?1012000
0110?2211011000100010001012100000110011020011111012101000310111110110?
??011011??0

Lariosaurus_calcagnii

000211011200000010011101000(01)0022121001000??00011001120100 20?101200
00110?2211011000100010010012100001010211020111110012111000311111111110
?00011011000

Lariosaurus valceresii

0002110112000000100110010001?00221(12)1001000??0?01100112????????01?000
011002?11011?00100010000012100000??10??10200111100121100003l??111121100
00?11?11000

Lariosaurus xingyiensis

00021101120001001001100000101?002212100100011 0?011001120?0000?101200001
1002211011?00100010011012100000000211020011110012111000 31??1100????100
011011000

Corosaurus

00001001?20000000000011 10000??0001000001001??01010110????0000??11100001
100120001000010011101011000010101010112000111001200002010011100101 01?
0?1100?010

Cymatosaurus

000211?10200101100(01)011(12)00000000(12)(23)0000010011?0?0(01)01100?0100
0011??200001100121001?0???0011?0?00????000???????????000000?????0????0111
00????1001?100?010

Wangosaurus

000210010200001000011000000000102311012100011 0?0101010?????????11?000?1
1002???01001??0001?0000110??????10???0?000?11001?0???00?????1??0110110?0
1001??0

Pistosaurus

0000210102?02?1?0100112000000102312012101?1?0112011???0?0000??????00011
001210110001?0011?1000????0?0??????113?00?1131220001??0????11??????1???1
00?1(12)0

Augustasaurus

0000210102???2?1?0100112000000100312012101?1?0012011201011000??01200000
10022101?001111011?000????00?00?00??103??02113102000120?????????????0?010
100???0

Yunguisaurus

00001101020010100001101000000??00312012101?1?0112010?020?10200011200001
10012(01)101002?110?1?1100?0000000??0??11311011131121001004002101100001
10001001110

Plesiosaurus
0000000102??2?1?00001000010001003120121010 1?0112010201011020??01000000
10012(01)010002011011010 00?1010?00100??1031101103112102000400210110000
?10100101110
Bobosaurus
00???(0
1)01000?00?0?1?1000120?000???????????0(12)?0?1?????????0021????????????01?
?01?

Part D. References

Benson, R., M. Evans, and P. S. Druckenmiller. 2012. High diversity, low disparity and small body size in plesiosaurs (Reptilia, Sauropterygia) from the Triassic–Jurassic boundary. PLoS ONE 7:e31838.

Cheng, Y.-N., X.-C. Wu, T. Sato, and H.-Y. Shan. 2012. A new eosauropterygian (Diapsida, Sauropterygia) from the Triassic of China. Journal of Vertebrate Paleontology 32: 1335–1349.

Holmes, R., Y. N. Cheng, and X. C. Wu. 2008. New information on the skull of *Keichousaurus hui* (Reptilia: Sauropterygia) with comments on sauropterygian interrelationships. Journal of Vertebrate Paleontology 28:76–84.

Jiang, D.-Y., M. W. Maisch, W.-C. Hao, Y.-L. Sun, and Z.-Y. Sun. 2006. *Nothosaurus yangjuanensis* n. sp. (Reptilia, Sauropterygia, Nothosauridae) from the middle Anisian (Middle Triassic) of Guizhou, southwestern China. Neues Jahrbuch für Geologie und Paläontologie, Monatshefte 2006:257–276.

Li, C., D.-Y. Jiang, L. Cheng, X.-C. Wu, and O. Rieppel. 2014. A new species of *Largocephalosaurus* (Diapsida: Saurosphargidae), with implications for the morphological diversity and phylogeny of the group. Geological Magazine 151:100-120.

Li, Q., and J. Liu. 2020. An Early Triassic sauropterygian and associated fauna from South China provide insights into Triassic ecosystem health. Communications Biology 3:63. doi: 10.1038/s42003-020-0778-7.

Lin, W.-B., D.-Y. Jiang, O. Rieppel, R. Motani, C. Ji, A. Tintori, Z.-Y. Sun, and M. Zhou. 2017. A new specimen of *Lariosaurus xingyiensis* (Reptilia, Sauropterygia) from the Ladinian (Middle Triassic) Zhuganpo Member, Falang Formation, Guizhou, China. Journal of Vertebrate Paleontology. doi: 10.1080/02724634.2017.1278703.

Lin, W.-B., M. Zhou, and D.-Y. Jiang. 2019. Systematic study of the eosauropterygians from the Triassic of South China. Science Press, Beijing, China, 154pp. [Chinese]

Lin, W.-B., D.-Y. Jiang, O. Rieppel, R. Motani, C. Ji, A. Tintori, Z.-Y. Sun, and M.

Zhou. 2021. *Panzhousaurus rotundirostris* Jiang et al., 2019 (Diapsida: Sauropterygia) and the recovery of the monophyly of Pachypleurosauridae. Journal of Vertebrate Paleontology. doi: 10.1080/02724634.2021.1901730.

Liu, J., O. Rieppel, D.-Y. Jiang, J. C. Aitchison, R. Motani, Q.-Y. Zhang, C.-Y. Zhou, and Y.-Y. Sun. 2011. A new pachypleurosaur (Reptilia: Sauropterygia) from the lower Middle Triassic of southwestern China and the phylogenetic relationships of Chinese pachypleurosaurs. Journal of Vertebrate Paleontology 31:291-302.

Liu, J., S.-X. Hu, O. Rieppel, D.-Y. Jiang, M. Benton, N. Kelley, J. Aitchison, C.-Y. Zhou, W. Wen, J.-Y. Huang, T. Xie, and T. Lv. 2014. A gigantic nothosaur (Reptilia: Sauropterygia) from the Middle Triassic of SW China and its implication for the Triassic biotic recovery. Scientific Reports 4:7142. doi: 10.1038/srep07142.

Ma, L.-T., D.-Y. Jiang, O. Rieppel, R. Motani, and A. Tintori. 2015. A new pistosauroid (Reptilia, Sauropterygia) from the late Ladinian Xingyi marine reptile level, southwestern China. Journal of Vertebrate Paleontology. doi: 10.1080/02724634.2014.881832.

Neenan, J. M., C. Li, O. Rieppel, and T. M. Scheyer. 2015. The cranial anatomy of Chinese placodonts and the phylogeny of Placodontia (Diapsida: Sauropterygia). Zoological Journal of the Linnean Society 175:415–428.

Rieppel O. 2001. A new species of *Nothosaurus* (Reptilia: Sauropterygia) from the upper Muschelkalk (lower Ladinian) of southwestern Germany. Palaeontographica Abteilung A 263:137-161.

Rieppel, O., and K.-B. Lin. 1995. Pachypleurosaurs (Reptilia: Sauropterygia) from the Lower Muschelkalk, and a review of the Pachypleurosauroidea. Fieldiana Geology 32:1–44.

Rieppel, O., P. M. Sander, and G. W. Storrs. 2002. The skull of the pistosaur *Augustasaurus* from the Middle Triassic of northwestern Nevada. Journal of Vertebrate Paleontology 22:577–592.

Wu, X.-C., Y.-N. Cheng, C. Li, L.-J. Zhao, and T. Sato. 2011. New information on *Wumengosaurus delicatomandibularis* Jiang et al. 2008 (Diapsida: Sauropterygia), with a revision of the osteology and phylogeny of the taxon. Journal of Vertebrate Paleontology 31:70–83.
